# Low-Frequency, Open, Sound-Insulation Barrier by Two Oppositely Oriented Helmholtz Resonators

**DOI:** 10.3390/mi12121544

**Published:** 2021-12-11

**Authors:** Yi-Jun Guan, Yong Ge, Hong-Xiang Sun, Shou-Qi Yuan, Xiao-Jun Liu

**Affiliations:** 1 Research Center of Fluid Machinery Engineering and Technology, School of Physics and Electronic Engineering, Jiangsu University, Zhenjiang 212013, China; gyjlxy@ujs.edu.cn (Y.-J.G.); geyong@ujs.edu.cn (Y.G.); 2 Key Laboratory of Modern Acoustics, National Laboratory of Solid State Microstructures, Department of Physics and Collaborative Innovation Center of Advanced Microstructures, Nanjing University, Nanjing 210093, China; 3 State Key Laboratory of Acoustics, Institute of Acoustics, Chinese Academy of Sciences, Beijing 100190, China

**Keywords:** sound insulation, low-frequency sound, open barrier, ventilation, Helmholtz resonators

## Abstract

In this work, a low-frequency, open, sound-insulation barrier, composed of a single layer of periodic subwavelength units (with a thickness of λ/28), is demonstrated both numerically and experimentally. Each unit was constructed using two identical, oppositely oriented Helmholtz resonators, which were composed of a central square cavity surrounded by a coiled channel. In the design of the open barrier, the distance between two adjacent units was twice the width of the unit, showing high-performance ventilation, and low-frequency sound insulation. A minimum transmittance of 0.06 could be observed around 121.5 Hz, which arose from both sound reflections and absorptions, created by the coupling of symmetric and asymmetric eigenmodes of the unit, and the absorbed sound energy propagating into the central cavity was greatly reduced by the viscous loss in the channel. Additionally, by introducing a multilayer open barrier, a broadband sound insulation was obtained, and the fractional bandwidth could reach approximately 0.19 with four layers. Finally, the application of the multilayer open barrier in designing a ventilated room was further discussed, and the results presented an omnidirectional, broadband, sound-insulation effect. The proposed open, sound-insulation barrier with the advantages of ultrathin thickness; omnidirectional, low-frequency sound insulation; broad bandwidth; and high-performance ventilation has great potential in architectural acoustics and noise control.

## 1. Introduction

Low-frequency sound insulation has always been a hotspot in the fields of acoustics and mechanical engineering due to its extensive applications in noise control, environmental protection, and architectural acoustics. Generally, porous and fibrous materials [1,2] and micro-perforated structures [3,4] are used to realize sound absorption, but their thicknesses are comparable to sound wavelengths, leading to relatively large sizes of absorbing structures.

In recent years, the emergence of metamaterials [5,6,7,8,9,10,11,12,13,14] and metasurfaces [15,16,17,18,19,20,21,22] have provided the feasibility to overcome the restrictions of these conventional materials of sound, and have been introduced to design several types of units of sound absorption. As examples, for Helmholtz resonators [23,24,25,26,27,28], the sound energy is absorbed through the cavity resonance and is dissipated around the neck by viscous friction; for sound membranes [29,30,31,32], the unit system is composed of a fixed elastic film and an object of different mass with asymmetric rigid platelets, and its resonant frequency can be adjusted by changing the film tension, finally realizing the absorption of low-frequency sound; for coiled Fabry–Perot resonators [33,34,35], the low-frequency sound energy is absorbed by thermal viscous loss in the coiling channel, and its frequency band can be adjusted flexibly by changing the channel length. In addition, other type of units, such as split-ring resonators [36]; ultrathin, metasurface-based structures [37,38,39,40,41,42,43,44]; and coherent perfect absorbers [45,46], can also be applied to sound absorption. Based on these types of units, the previously designed structures of sound absorption usually have the advantages of subwavelength thickness and high efficiency. However, in some practical applications, it is not only necessary to realize sound insulation, but also requires ventilation on both sides of the structure. 

To overcome this, researchers have been devoted to designing open, sound-insulation systems based on different physical mechanisms, which mainly include open sound absorbers based on the coupling of resonances [47,48,49]; open sound silencers by destructive interference [50,51]; ultra-sparse, sound-insulation walls based on artificial Mie resonances [52]; and unidirectional, open, sound-insulation channels by asymmetric multiple scatterings [53]. Beyond that, based on the generalized Snell′s law [54], acoustic metacages [55], open sound tunnels [56], and window structures [57,58] have also been realized by designing acoustic metasurfaces with the desired gradient-index profiles. These open structures can realize sound insulation and ventilation simultaneously. However, it is difficult to obtain high-performance ventilation and broad working bandwidth owing to their complex structures and multiple design mechanisms; the design of open structures with simple configuration, broadband sound insulation, and high-performance ventilation still poses a challenge.

In this work, we propose a low-frequency, open, sound-insulation barrier composed of a single layer of periodic subwavelength units, where each unit consists of two identical, oppositely oriented Helmholtz resonators. Based on sound reflections and sound absorptions under the excitation of the coupling of symmetric and asymmetric eigenmodes of the unit, we realized low-frequency sound insulation with a minimum transmittance of 0.06 at 121.5 Hz. Additionally, we discussed the influence of the parameter *w* on the performance of sound insulation, and observed broadband sound insulation with a fractional bandwidth of 0.19 by increasing the layer numbers in the open barrier. The measured and simulated results agreed well with each other. Finally, the application of the multilayer, open barrier in designing a ventilated room with an omnidirectional, broadband, sound-insulation effect was discussed in detail.

## 2. Design of the Model and the Prediction Method 

**Figure 1:**
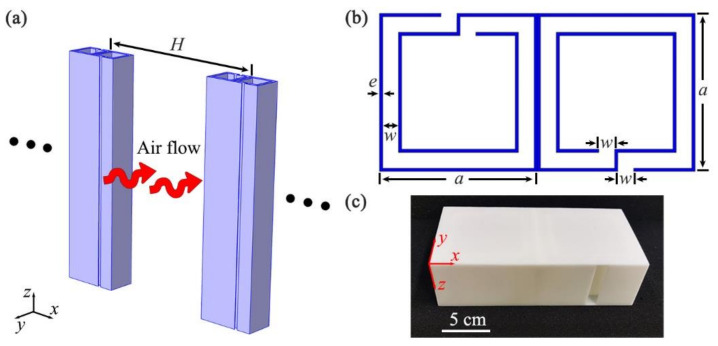
(**a**) Schematic of an open, sound-insulation barrier; (**b**) cross-sectional view of a unit; (**c**) a photograph of the unit.

As schematically shown in Figure 1a, we designed an open, sound-insulation barrier composed of a single layer of periodic units, in which *H* was the distance between two neighboring units. Figure 1b shows the cross-sectional view of the unit constructed by two identical, oppositely oriented Helmholtz resonators, which were composed of a central square cavity surrounded by a coiled channel. The parameters *a*, *e*, and *w* were the length of the resonator, the thickness of the wall, and the width of the channel, respectively. The unit was fabricated with epoxy resin based on 3D printing technology, and its photograph is shown in Figure 1c. Here, we introduced the software of COMSOL Multiphysics to numerically simulate the characteristics of sound insulation. In the simulations, the module of Thermoviscous Acoustic-Solid Interaction was used inside the unit, but the module of Acoustic Pressure was adopted outside the unit, owing to the huge computation load. The thermoviscous-acoustic boundary layers were applied to the surfaces in the unit, and the thermoviscous-acoustic coupling boundary was used for the interface between the channel and the external space. In the numerical models, these parameters were selected as *H* = 400 mm, *a* = 100 mm, *e* = 2 mm, and *w* = 10 mm. The material parameters of epoxy resin were the density $\rho_{e}$= 1180 kg/m^3^, the longitudinal wave velocity $c_{l}$= 2720 m/s, and the transversal wave velocity $c_{t}$= 1460 m/s. Additionally, the parameters of air could be calculated as $\rho_{0}=p_{0}M/RT$ and $c_{0}=\sqrt{\gamma RT/M}$, in which the ratio of the molar heat capacities γ, the molar mass *M*, the air temperature *T*, the molar gas constant *R*, and the air pressure $p_{0}$ were 1.4, 28.97 × 10^−3^ kg/mol, 293 K, 8.31 J/(mol/K), and 101.325 kPa, respectively.

## 3. Simulated Results and Discussion

### 3.1. Performance of the Open, Sound-Insulation Barrier

**Figure 2:**
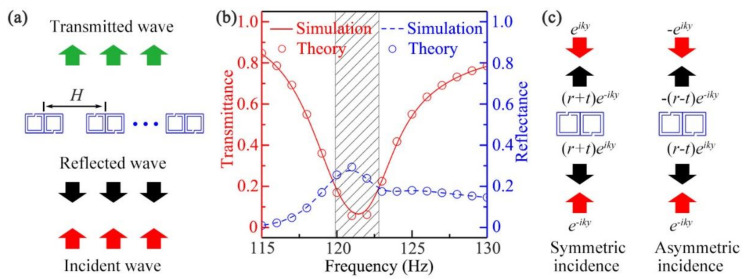
(**a**) Schematic of the performance simulation of the designed open, sound-insulation barrier; (**b**) the transmittance (red solid line for simulation and red open circle for theory) and reflectance spectra (blue dashed line for simulation and blue open circle for theory) of the open, sound-insulation barrier created by a normal incidence of sound; (**c**) schematic of symmetric and asymmetric incidence.

Figure 2a shows the schematic of the performance simulation of the designed open sound barrier, in which the incident wave was set to normal incidence in free space. The simulated transmittance (red solid line) and reflectance (blue dashed line) spectra are illustrated in Figure 2b. We could see that in the range of 119.9–122.8 Hz (black shaded region), the transmittance was lower than 0.2 and the minimum value was approximately 0.06 at 121.5 Hz. However, it was worth noting that the corresponding reflectance was only about 0.26, indicating that most of the sound energy was absorbed by the barrier. On the other hand, the transmittance and reflectance of the barrier were also calculated theoretically. We defined *t* as the transmission coefficient and *r* as the reflection coefficient, considering that the incidence from one side could be decomposed into a superposition of the symmetric incidence and asymmetric incidence [45], the corresponding reflection coefficients $r_{s}$ and $r_{a}$ could be described as $r_{s}=r+t$ and $r_{a}=r-t$ [47,59], respectively (shown in Figure 2c). We could then obtain $r=\left(r_{s}+r_{a}\right)/2$ and $t=\left(r_{s}-r_{a}\right)/2$. The calculated transmittance ${\left|t\right|}^{2}$ (red open circle) and reflectance ${\left|r\right|}^{2}$ (blue open circle) are also shown in Figure 2b, and it was obvious that the theoretical results agreed well with the simulated ones. In addition, it was noted that the thickness of the barrier was 100 mm (about 1/28 wavelength), and the distance *H* was twice as large as the unit width (2*a*), showing the sub-wavelength thickness and efficient ventilation of the barrier.

### 3.2. Mechanism of Sound Insulation for the Open Barrier

**Figure 3:**
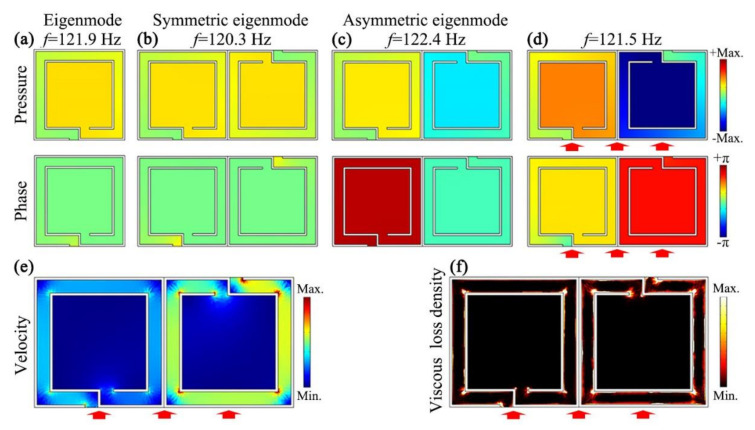
(**a**) Simulated pressure and phase eigenmodes of a single Helmholtz resonator at 121.9 Hz, and those of the unit, corresponding to the following: (**b**) symmetric eigenmode at 120.3 Hz; (**c**) asymmetric eigenmode at 122.4 Hz; (**d**) simulated pressure and phase distributions in the unit created by the normal incidence of sound (red solid arrows) at 121.5 Hz. Simulated distributions of (**e**) velocity of air flow and (**f**) viscous energy-loss density in the unit created by the normal incidence of sound (red solid arrows) at 121.5 Hz.

Next, to provide an insight into the mechanism of sound insulation, we simulated the pressure and phase eigenfunctions of a single Helmholtz resonator and a single unit, approximately 121.5 Hz, which are shown in Figure 3a–c. As shown in Figure 3a, there only existed a single eigenmode at 121.9 Hz for the Helmholtz resonator. However, for the unit, we could observe two types of eigenmodes at 120.3 Hz and 122.4 Hz (Figure 3b,c), which were denoted as symmetric and asymmetric eigenmodes based on their field distributions. Here, for the symmetric eigenmode, the pressure and phase distributions in the two resonators were the same, and were the same as those of the Helmholtz resonator at 121.9 Hz. However, for the asymmetric eigenmode, the corresponding phase distributions in the two resonators were opposite. Based on these results, it was deduced that the excitation symmetric and asymmetric eigenmodes of the unit were caused by the reverse placement of two Helmholtz resonators. 

Figure 3d shows the pressure and phase distributions of the unit excited by the normal incidence of sound at 121.5 Hz. It was observed from the pressure distribution that the characteristics of the excited mode were close to those of the asymmetric mode, but the sound energy inside the central cavity was relatively stronger. Such a phenomenon indicates that, in addition to a small amount of sound reflection, another part of sound energy was absorbed inside the unit by the excited mode. However, it was worth noting that the phase difference of the two resonators was between those of the symmetric and asymmetric eigenmodes. Therefore, we deduced that the excited mode at 121.5 Hz was attributed to the coupling of the symmetric and asymmetric eigenmodes. Moreover, we simulated the velocity and viscous energy loss density distributions of air in the unit at 121.5 Hz, which are shown in Figure 3e and Figure 3f, respectively. We could see that the velocity of airflow along the channel of the right resonator was higher than that of the left resonator, and so was the viscous energy loss density on the inner wall of the channel. This was because the difference of pressure amplitude between the two ends of the coiled channel (Figure 3d) in the right resonator were larger than that in the left resonator. Therefore, we demonstrated that the sound insulation of the open barrier arose from both sound reflections (Figure 2b) and sound absorptions by the excited mode, and the absorbed sound energy propagating into the central cavity was greatly reduced by the viscous loss in the channel.

### 3.3. Bandwidth Optimization of the Open, Sound-Insulation Barrier

**Figure 4:**
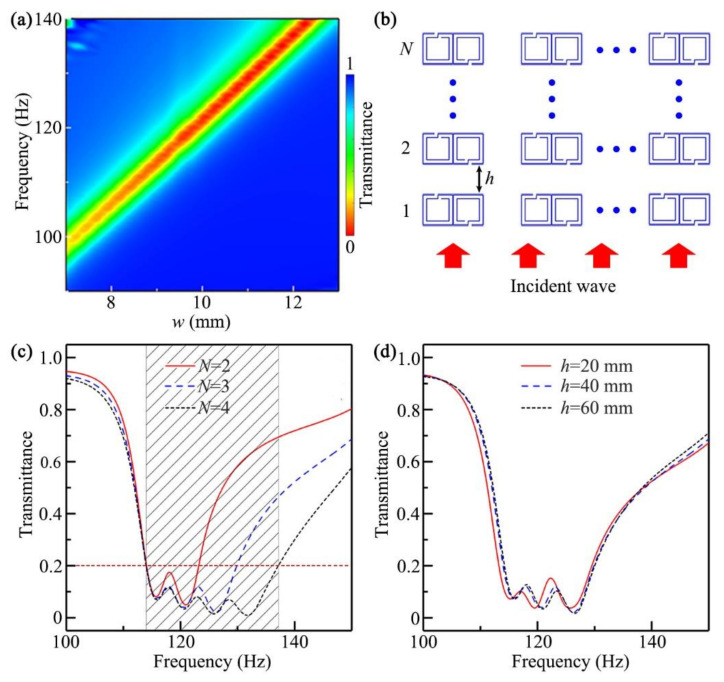
(**a**) Simulated transmittance spectra through a single-layer, open barrier with different values of *w*; (**b**) schematic of a multilayer, open barrier composed of *N*-layer units; (**c**) simulated transmittance spectra of the multilayer barriers with *N* = 2 (red solid line), 3 (blue dashed line), and 4 (green short dashed line); (**d**) simulated transmittance spectra of the barriers (*N* = 3) with *h* = 20 (red solid line), 40 (blue dashed line), and 60 mm (green short dashed line).

Figure 4a shows the simulated transmittance spectra through the unit with different values of *w*, in which the other parameters were the same as those in Figure 2b. We could see that, with the decrease in the value of *w*, the working band for the low transmission of sound shifted to the low-frequency region. Thus, we could modulate the working band of the open barrier by simply adjusting the parameter *w*.

Based on the aforementioned results, we discussed the optimization of the working bandwidth of the open barrier. To realize it, we introduced a multilayer open barrier composed of *N*-layer units, and its configuration is shown in Figure 4b, in which *h* is the distance between two adjacent layers. Figure 4c shows the simulated transmittance spectra of the multilayer, open barriers in free space with *N* = 2, 3, and 4, in which the parameter *w* of the *N*-layer unit was $w=9.3+0.7\times \left(N-1\right)$ mm, *h* = 40 mm, and the other parameters were the same as those in Figure 2b. We found that the bandwidth increased gradually with the increase in the number of layers, and the transmittances were below 0.2 in the range 114.0–137.2 Hz for *N* = 4 (black shaded region), indicating that the fractional bandwidth (the ratio of the bandwidth to the center frequency) could reach about 0.19. Thus, we could effectively increase the working bandwidth by introducing the multilayer, open system. Compared with previous work [52], the distance between two adjacent units of the multilayer, open sound barrier in our work was the same as that in Figure 2, indicating that the bandwidth optimization did not affect the performance of ventilation. 

Furthermore, we investigated the influences of the parameter *h* on sound insulation. Figure 4d shows the simulated transmittance spectra of the multilayer, open barrier (*N* = 3) with *h* = 20, 40, and 60 mm, in which the other parameters were the same as those in Figure 4c. We could see that there existed a slight difference between the spectra with *h* = 20 and 40 mm, and the spectra were almost the same when *h* was larger than 40 mm, which indicated that the sound insulation of the multilayer, open barrier had little to do with the distance *h*.

### 3.4. Ventilation Optimization of the Open, Sound-Insulation Barrier

**Figure 5:**
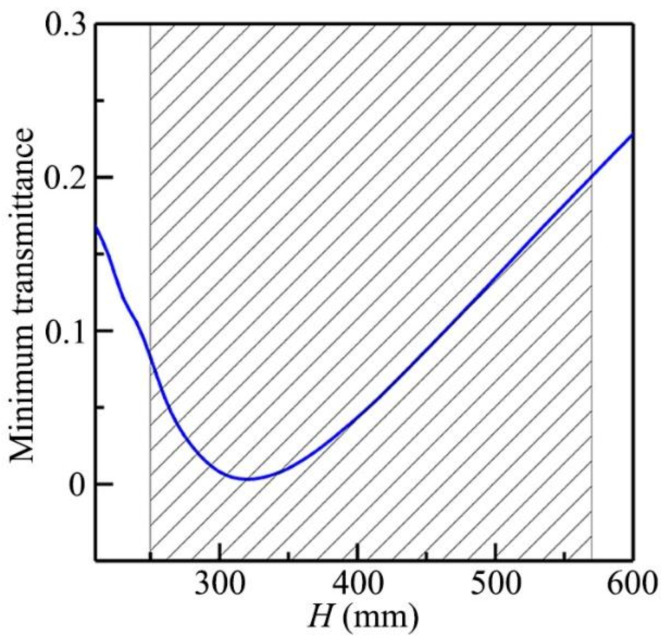
Simulated minimum transmittance spectrum through the open barriers with different values of *H*.

The ventilation characteristic of the open barrier was mainly dependent on the distance *H*. Figure 5 shows the simulated minimum transmittance spectrum through the open barriers with different values of *H*, in which the other parameters remained the same as those in Figure 2b. We found that the minimum transmittance could reach about 0.002 at *H* = 320 mm, and it was lower than 0.2 in the range of 250–570 mm (black shaded region), showing high-performance sound insulation with ventilation of the proposed open barrier.

## 4. Experimental Verification

### 4.1. Measurement Set-Up

**Figure 6:**
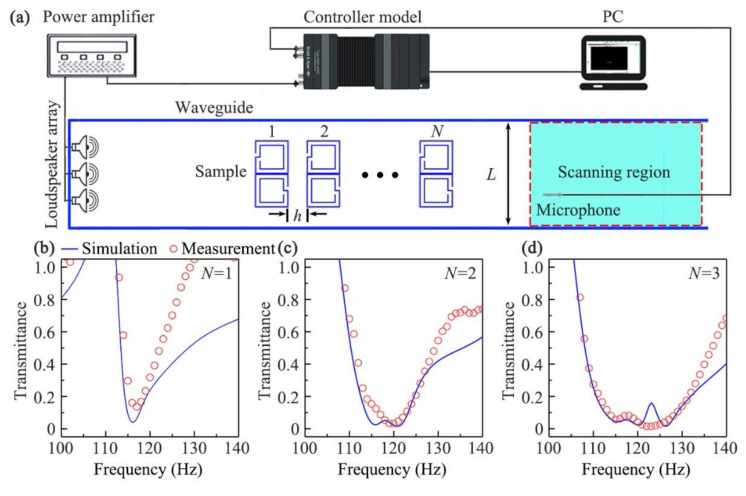
(**a**) Measurement set up. Simulated (blue solid line) and measured (red open circles) transmittance spectra through the sample composed of *N*-layer units in the waveguide, and the corresponding layer number: (**b**) *N* = 1; (**c**) *N* = 2; (**d**) *N* = 3.

To demonstrate the sound-insulation performance of the open barrier, we simulated and experimentally measured the transmittance spectra through the sample in a straight waveguide (shown in Figure 6a), in which the width of waveguide (*L*) was the same as the parameter *H* in Figure 1a. The sample composed of *N*-layer units was placed at the middle position of the straight waveguide with a size of 2 × 0.4 × 0.06 m^3^, which was made of acrylic plates to satisfy the sound-hard boundary condition. A loudspeaker array, driven by a power amplifier, was placed at the left entrance to generate incident sound signals, and a 0.25-inchmicrophone (Brüel & Kjær,Nærum, Denmark., type-4954) was used to detect transmitted sound signals in the scanning region from the open right entrance. The measured data were recorded by the Brüel & Kjær 3160-A-022 module, and were analyzed by the software PULSE Labshop. The transmittance spectra were calculated as the ratio of the results with and without the open barrier.

### 4.2. Experimental Results

Figure 6b, Figure 6c, and Figure 6d show the measured (red open circles) and simulated (blue solid line) transmittance spectra through the sample in the waveguide with *N* = 1, 2, and 3, respectively, in which the parameters *L* = 400 mm, *h* = 40 mm, and the other parameters were the same as those in Figure 4c. We could see that the measured results agreed well with the simulated ones in Figure 6b–d, and the bandwidth of sound insulation obviously increased with the increase in the number of layers *N*, which was consistent with that in Figure 4. Therefore, we experimentally demonstrated the performance and bandwidth of sound insulation for the designed open barrier.

## 5. Application of the Open, Sound-Insulation Barrier

**Figure 7:**
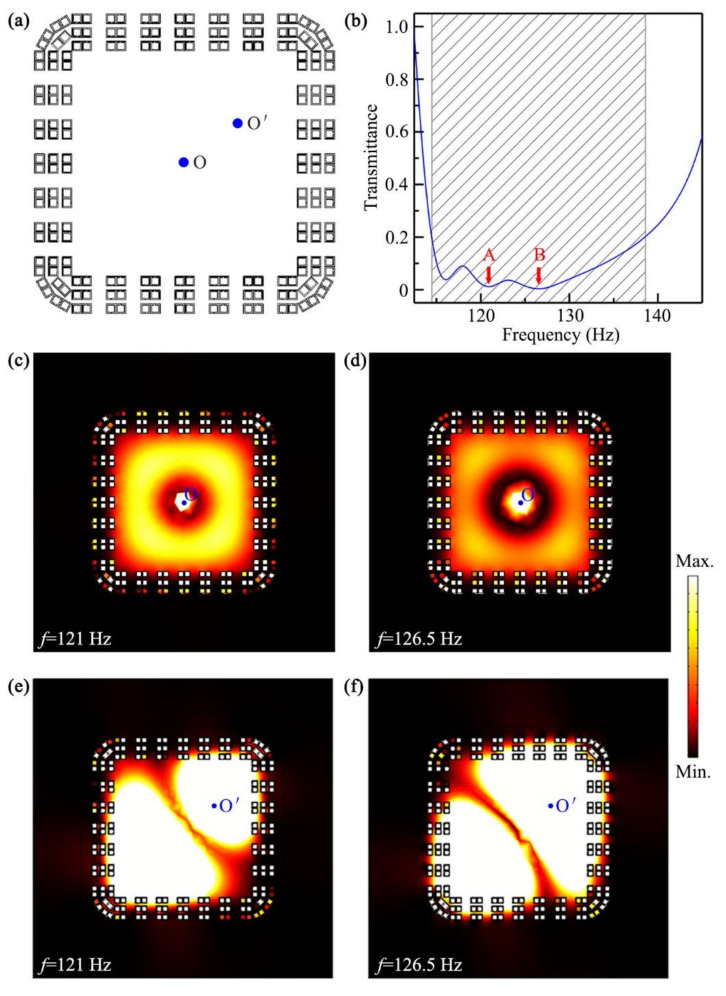
(**a**) Schematic of a ventilated sound-insulation room with a cylindrical sound source at the point O or O′; (**b**) simulated transmittance spectrum through the room created by the sound source at the point O. Intensity distributions in the ventilated, sound-insulation room at (**c**) 121 Hz and (**d**) 126.5 Hz created by the sound source at the point O, corresponding to the points A and B in (**b**), respectively. Intensity distributions in the ventilated, sound-insulation room at (**e**) 121 Hz and (**f**) 126.5 Hz created by the sound source at the point O′.

Finally, we designed a ventilated, sound-insulation room based on the multilayer, open barrier in Figure 4c with *N* = 3, and the configuration is shown in Figure 7a. Figure 7b shows the simulated transmittance spectrum through the sound-insulation room, in which a cylindrical sound source was placed at the center (point O). We found that the transmittances were lower than 0.2 in the range 114.5–138.6 Hz (black shaded region), showing the fractional bandwidth of 0.19. To obviously exhibit the performance of sound insulation, we simulated the intensity distributions in the sound-insulation room at 121 Hz and 126.5 Hz, created by the sound source at the point O (shown in Figure 7c,d), which corresponded to the points A and B in Figure 7b, respectively. We could see that, for both frequencies, the sound energy in all directions was almost insulated totally by the open sound barrier. Beyond that, to verify its robustness, we simulated intensity distributions in the ventilated, sound-insulation room created by the sound source at the point O′ (shown in Figure 7e,f), and the simulated results also present high-performance sound insulation, which was almost the same as that in Figure 7c,d. Therefore, the proposed sound barriers had the advantages of broad bandwidth, high-performance ventilation, and omnidirectional sound insulation, and showed great potential for applications in architectural acoustics and noise control.

## 6. Conclusions

In conclusion, we demonstrated a type of broadband, low-frequency, open, sound-insulation barrier. Based on the coupling of symmetric and asymmetric eigenmodes of each unit, low-frequency sound insulation through the open barrier could be obtained around 121.5 Hz with a minimum transmittance of 0.06, which arose from both sound reflections and absorptions, and the absorbed sound energy was reduced greatly by the viscous loss in the coiled channel. Moreover, the distance between two neighboring units *H* was twice as large as the unit width, showing high-performance ventilation. Additionally, we discussed the influence of the parameter *w* on the sound insulation, and obtained the working band of sound-insulation shifts to the low-frequency region with the decrease in *w*. Based on this, we realized broadband sound insulation with a fractional bandwidth of 0.19 by increasing the number of layers in the open barrier. Moreover, we experimentally measured the performance of open, sound-insulation barrier in a waveguide, and the measured results agreed with the simulated ones. Finally, we discussed the application of the multilayer, open barrier in the design of a ventilated, sound-insulation room in detail, which demonstrated the potential application of omnidirectional, broadband sound insulation for the barrier. Therefore, the proposed low-frequency, open barrier with the characteristics of omnidirection, broadband sound insulation and high-performance ventilation provides diverse routes to design advanced, sound-insulation structures in noise control and architectural acoustics.

## Data Availability

Data is contained within the article, further inquiries can be directed to the corresponding authors.
